# Discussion

**Published:** 1914-01-15

**Authors:** D. M. Cattell


### DISCUSSION.

BY DR. D. M. CATTELL.

Mr. President: The first thing I want to do is to make a suggestion that this Association has not been in the habit of doing, and that is that its Essay Committee urge upon the writer of the paper to allow the man who is to open the discussion upon that paper to have a copy of it, that he may look it over. In this present instance I had never heard the paper before. How can I, or any one, discuss the paper intelligently, unless he has had at least a few minutes time to study the question, unless that particular question be one of his hobbies, something that he is very familiar with, and has already studied

Now let me make another proposition,—that it is just as easy to teach a man to ride a bicycle through a correspondence school as it is for the profession to make much headway without research work. I would like to pay my respects, if I knew how and had a good opportunity to do so, to those poor, misguided minds that are so much opposed to the medical and dental profession studying anatomy, studying through the living brute, may be, how to prevent death in the human being. I have a dog somewhere living that I think much of. I will fight the man that abuses him, and I will fight for all that is in me for my dog. Nevertheless, I will sacrifice that dog—a dozen times if it were possible—if, through the study of that animal and others I may save my boy's life.

Now research work as a specialty we used to know nothing of, and we knew but little of its value, but as research work develops, in spite of the poor, misguided individuals that cry out against it, as that work increases in quantity so do we increase our knowledge of how to save human life. So from that standpoint I am strongly in favor of such research work. Oh, that Mr. Carnegie were interested in the dental world, or someone else that had the millions to spend. Such millionaires are doing great work in turning over their millions to the study of things that pertain to the physician, but the dental world needs research laboratories too. It needs well equipped men that have made dentistry a life study, men who are interested in oral surgery rather than in general surgery. For such men to work alone, without help, without laboratories, is all but impossible, so I say again, if we could but have a Rockefeller, or a Carnegie, so to speak, some one that will equip our laboratories, and pay our eminent men for their time in this research work, how much more rapidly we would gain, how much greater we would grow.

The particular points in the paper in which I was interested as it was read, was the appeal to the profession for funds. I can not say much upon the paper, except to compliment and assure you that I believe in research work. I know we are not doing enough of it, I believe we would be doing more but we are handicapped by the lack of men with money to equip these laboratories. There is no incentive given to our young men of today that are leaving college to take up such work; but if we had well equipped laboratories for them, if there were such in existence today, many young men that have just finished their collegiate courses would be willing, glad, to take up the work, and further it along.

We have in our profession many eminent men, worthy to be called to this research work, but the laboratories can not be furnished them, the means of existence while they are at work can not be assured them, and they will not enthuse even at the call of suffering humanity. There are so many things, so many calls upon the individual, so many calls upon the Dental Association, for appropriations; money for this, for that and many good things.

A grand thing was instituted through the efforts of our brother, Noel, to relieve indigent dentists. It is one of the most notable things that the profession as a whole has ever taken up. It does seem to me, as suggested by the essayist that if every man in the profession would give a dollar a year, we could equip a few laboratories, and we could have two or three men working day after day, finding out some of the hidden things. I remember two years ago the Chicago Dental Society raised $700.00, and we thought it was a big amount, some objected because it was to be used to study about erosion. Well, we only worked about four months, and the $700.00 was exhausted and they had not gotten anywhere. The workers gathered together the available data, and they tried out some experiments that had been devised before with no results. They left us nothing but one suggestion, and that was to treat the mucous membrane instead of the teeth. Now if $700.00 only did so much, we shall need seventeen times $700.00 to equip proper laboratories that the entire line of research work shall go on. I stand ready, and always have been ready, as an individual, to give my little quota to establishing and helping along such research movements.

We need a Johns Hopkins University, if you will, as a starting point, somewhere for the dentist. When teachers in the medical world, in the medical schools, have a vacation they go to Europe,—they may go to Vienna, to Berlin, or they may go to Johns Hopkins and take a special course in the research laboratory, or they may go to Chicago, in the great University laboratory there. Where do the dentists go when they take their vacations? They go to the woods. It is time that some of them went to Johns Hopkins. It is time that some of the dentists that have some knowledge of bacteriology, pathology, and so on, went along further, and get something that they can bring back to the profession. Why don't they do it? Because the laboratories there are not equipped for dental research work. To equip a laboratory properly would require a large amount of money, and also more money for the men who shall do the research work. Equip a laboratory that belongs to the profession, equip one that we can call our own, maintain it as it should be maintained, and we shall have not one, but half a dozen or more, of our brightest and best young men willing to give their time, their energy and their very best endeavor to such a cause as is ours.

BY DR. L. G. NOEL.

I received a message through Dr. Cottrell, saying Dr. Melendy wanted me to say a word or two on this subject, in support of the Committee. I am sorry indeed that Dr. Melendy is not here, or Dr. Weston A. Price, or at least one of the members of that Committee, to speak to you on this important subject, because it is a most important one, and I feel that I am not competent to present it to you in such a manner as to enthuse you as I should like to see you enthused, or arouse you as I should like to see you aroused on this subject. And then, too, as Dr. Cattell has remarked, I have annoyed you a good deal lately by suggesting something you might do with your money, and I dislike to appear before you again so soon after your extreme kindness to me, because the Tennessee Dental Association and the Colorado Societies are the only ones that have been so good to me. But the matter is important, and I come to tell you merely what I have resolved myself to do. I have resolved to contribute my little mite to this matter, as I have done to the other matter, and as I expect to continue doing. We have only to pause and reflect how little we really know, to realize that we stand like the boy upon the shore, who has just gathered up two or three pebbles. We have only to look at the long stretch of beach, and then let our minds run along achievement, to realize that we have only picked up a few pebbles on the sea-shore of truth, and that we are yet groping for something. Engaged, as we are, in a practice that absorbs all our time, what can we do in the matter of research work, encumbered as we are with the labor of our daily practice? Nothing. It is, perhaps, only two or three men that are produced within a century that can do anything at all under our present circumstances,—probably only three men have been produced in the last century that have done very much. We have produced in America our G. V. Black and W. D. Miller, and J. Leon Williams. We perhaps could not think of more than three or four men, throughout the world, who, in the last century, have done very much in the matter of research work—while supporting themselves by a dental practice.

What we need to do is to educate young men in these laboratories who are bright, and who have scientific minds, and naturally take to this kind of work, and give promise of success with this kind of work, and let them give all of their time to it.

We want, of course, some great benefaction, like that which John D. Rockefeller has given to the medical profession; we want such men as Rockefeller and Carnegie to do something for the dental profession—but are they going to do it unless we show that we are in earnest about it, and are doing something ourselves? Not much. Then let us arouse ourselves and contribute our mite to this cause. If we could only have two or three such men as W. D. Miller and G. V. Black engaged in such a laboratory as we contemplate in this movement, who do not have to give a moment's time to the preparation of their daily bread, or a minute's thought to finances, but give all of their time to searching out these questions, it may be that we shall know something about oral sepsis. We should know something about the causes of cancer, and it is just as likely that the dental profession will work out such problems as it is that the medical profession will. We have done something along the line of anesthesia. I tnink the world will admit this. Where would the world be today had it not been for the dental profession? And if we had such minds as Black and Miller at work, young men, giving their lives and time, untrammeled by professional cares, to this work, we might work out the cancer question. They are not getting to it in the Johns Hopkins laboratory very fast. Nobody has yet found out the cause of cancer. Somebody bobs up every day or two with a new theory. I noticed in the paper yesterday some Dutchman that thought he had found the parasite that caused it. Whether it is a parasite or abnormal cell growth—heaven knows, we see enough of it in the human mouth. The world at large does not know very much about oral sepsis. And that brings us right back to the subject of Dr. McRae's paper. I wanted very much to say something on that subject, but I felt it was too big a subject for a little man like me to talk about, but it is not generally known that carious teeth carry nearly all of the microorganisms that we know anything about. If the general public knew that fact do you suppose they would let children's teeth melt away like snow in the sun, and let those little mouths remain full of those deadly germs from the time they are two years old—No, they would watch these teeth, and when cavities come in them, they would have them filled, and the mouth would be properly treated, and that infection cleaned out, I think they would. They certainly attend to the blooded horse and to the blooded cow, and even to the blooded dog—the lower animal is properly attended to, and it is merely ignorance of these subjects that keeps the general public from being aroused thoroughly.

But they are awakening. Every day we see an editorial in some great medical journal on the subject of oral sepsis, and some such man as Hunter or Landon bobs up, and we get very much offended because he calls our attention to the fact that we have been cultivating foci of infection in the human mouth by badly fitted crowns and bridges, and utilizing diseased teeth for such abutments, and we get awfully offended because he calls attention to that, but really he is doing the dental profession more good than any of our own writers. It is the physician that can put his finger on the sore spot that is likely to benefit his patients.

I wish I could talk to you on this subject like Price. I tried my best to get him down here. Dr. Winn and I attended a meeting in March, and we had the pleasure of meeting Price and Burkhart, and they promised to try to get down here and talk on their favorite subject, but since I have been back I have had letters from Dr. Price in which he writes me to speak for him, as he said it would be impossible for him, on account of his various engagements to get here, which he regretted, but that Melendy and the other Committeemen would be present. If you could have heard Dr. Price, you would have been aroused like myself. His idea is that we should do our best, in the hope that some great man like Rockefeller or Carnegie will come along and and help us out. We are going to have great laboratories in connection with the great medical school that Carnegie has given so much to, and I am happy to tell you that the Dental Department at Vanderbilt will benefit. The newspapers have kept that matter in the dark and you don't know it unless you have been talking to Dr. Morgan, or somebody else that knows about it. We want a great laboratory of our own. Will you not do something to aid? I am going to do my little.

### DR. J. D. TOWNER.

I want to arraign the dental profession—being one of you myself I feel I can have that privilege.

I heard Dr. Morrison state a few moments ago upon the floor that the general public was very slow to do this and to do that. My opinion is that the general public is waiting, eager, anxious, expecting something to happen, and the dental profession is the individual and the body that is slow.

Now, let's see if this is not so. We have been discussing this matter of oral hygiene for years and years. I have missed but one meeting of the Tennessee Dental Association since I have been a member, about fifteen years, and I have heard it every time I have been present. Yet what has been done? Not one thing. You haven't even put a little squib in your newspaper to the effect that dirty mouths were prevalent in the community in which you live, and that dirty mouths would cause disease, and that certain things were found in the mouth. You have not told your people a thing, and it has been for one of two reasons. One is that you don't know anything yourself; and the other is that you are too indifferent about it to make any well directed effort. Now I may be wrong, but I don't think I am. I can't figure it out any other way.

We are in our offices and a patient comes in. "Doctor, I want this thing done thus and so." We jump around as though our patient was ex-president Roosevelt, or some other equally celebrated personage, and do what we are told to do, and when we are asked to do it, we dismiss and thank the patient and tell him to call again. That is the average dental practice today. It is so in every office, no matter where you practice, whether in a small place or the city,—it is the same thing. It is the tendency of the dental profession. Until we get away from this subserving and assert some independence, and let the public know two things,—first, that we know exactly what we are doing, and how and when to do it, and that we are going to do it as we think best, regardless of what they think about it; second, that we educate that person, which is just as much a part of our obligation as it is to relieve them from suffering. I hold that you do not fully discharge your obligation to a patient until you have given that patient full and complete instructions that will enable him to properly care for his teeth with the least possible amount of assistance from you. This is as important, and as much a part of your obligation, as doing the work itself.

Much can be said along this line to show you that it is the dental profession that is indifferent, and not the public. Not until we get away from this subservient idea ourselves, and educate the community in which we live to the fact that we are not mere mechanics, shall we progress, and get that recognition that we are striving for and fully entitled to have. Say what you will, no mechanic, no matter how good he is in any of the different branches of mechanics, ever rose to any great amount of prominence in cultivated communities, who has, as his objective only, the pay-roll.

When you shall have put dentistry, which is a part of the healing art, and which is a part and parcel of medicine, down on a par with the cabinet maker, the automobile repairman, and the blacksmith, you are doing yourself an injustice, and you are doing the public among whom you live an injustice also. Now how are you going to get away from this situation?

We must all realize, that the greatest strides in dentistry will be made in the line of the prevention of disease. You must realize again that the greatest part of dentistry is that part which pertains to medicine. A filling is nothing in the world but a stopper driven into a hole to prevent the ingress of something. That is all you can make out of it, no matter how beautiful it all is. We must find how we can give the greatest amount of immunity to disease. That is the point to which we must work,—how we can make dental caries impossible?

Not until we get our laboratories, and get men capable of doing research work, can we tell how to do these things. In the mean time, we can educate the public, and let them know what we are doing; and that will help some.

If I had not conceived the idea, some fifteen or more years ago, that the greatest part of dentistry was that part of it which was relatively medical, I would not be talking to you today in the capacity of a dentist. My idea was to study medicine, to go into surgery, because I liked it, and the turning point of my career was the fact that a number of operations in which I assisted proved fatal, and I came to the conclusion that a man of my temperament had better have the life of a tooth intrusted to him than the life of an individual, considering that I could do a great deal in a medical way by competent, intelligent treatment of the mouth in a medical way, and that is my practice.

We must let the public know that we are investigating, and until we do that we need not hope to advance one step further as a profession. We may have this man and that man ranking high in some line of practice, but as a profession we are not advancing . You need not, as Dr. Noel says, expect any man to help you unless you convince him that you are going to help yourself, especially if you are depending upon Carnegies and Rockefellers to give you money, because I have been up against Mr. Andrew Carnegie myself, and I know what it means. My pocket-book is just as empty now as it was when I approached him. You must prove beyond a question of doubt that you are doing something to help yourself, and then you will get assistance, and not until then.

I believe in research. I believe the general welfare of the dental profession, and of the public to whom we minister, depends upon research. That is the foundation, I think, of future advance in dentistry. I believe we all should not only encourage the present project with our finances, but in every other way that it is possible for us to do.

Just think, now, if you please,—how many dentists do you know outside of Miller, Black and Williams that have really given the profession anything of any consequence? We have had good technical things given to us, but who has done it? It has been the dental supply houses—the commercial element that is in the profession. They have established laboratories; they have grown clinics; they have done this and that in research work, and they have given it all to us, and we have gobbled it up. Every time anything good comes around that gives us an idea or that promises to be of advantage, we buy it even though a dentist has not produced it, and not only the dentist has not done it, but he really does not appreciate the effort and expense that is required,to thrash these things out and develop them into a certain form which can be used by the dental profession. It is the commercial side of dentistry, the business men catering to the needs of the dentist, which has given the dental profession one of its greatest advantages.

Now we need laboratories to correct this impression upon the public, this impression upon the profession. We need laboratories to take these things that are given us, and analyze them, and if possible, improve upon them.

DISCUSSION CLOSED BY A. J. COTTRELL.

I don't know whether you are all weary or not, but Dr. Melendy is a member of this Committee of the National Dental Association on this research work, and his main object in presenting the paper was to try, if he could, to present the importance of that work to this body, and if possible to arouse the interest and co-operation, not of the Association as a body, but of the individual members of the Tennessee State Dental Association, in this work.

The effort of this Committee, and the effort of Dr. Melendy, has been directed towards the one end of trying to impress upon each member of the profession the great good that would accrue to the profession as a whole if we take up this work, and give it the aid that it deserves.

He states these needs of the profession, a few of them, specifically, to call your attention to the fact that there are a number of things that we are brought in daily contact with that we have never understood, a number of tangles that have never been unraveled. It was not his desire so much to furnish you a paper that would enlighten you as to arouse interest in this subject on the part of members of this body.

There is no doubt in my mind that this work will be taken up and carried out, and the extent to which it will be carried out depends simply and entirely upon the efforts of the individual members of the profession.

Dr. Cattell has just told us of the Chicago Dental Association giving $700.00 and that it didn't go very far. Now, gentlemen, it remains with you and me and the other common, ordinary little fellows, as to just how far this work is going,—$700.00 worth, or 70 cents worth, or to the point of being successful.

The attitude of many of our dentists reminds me of the fellow who wanted to be humane, and suggested that the dog's tail be cut off just a little bit at a time—that seems to be pretty much the idea with a great many of us when it comes to doing the work which is for the benefit of the profession as a whole. We try to cut off the dog's tail just a little bit at a time, and it generally depends on some other body to do it.

The point the essayist wanted to impress on you is that this is your work, yours and mine. It is something we cannot, in honor to ourselves, leave or delegate to somebody else.

You know that the people in this section of the country have a great habit of leaving everything of this kind for the fellows "down East," as they have plenty of money, and better educational facilities, so they leave it to them; but we want to impress upon you that you owe your Association a duty, and that this is an opportunity to discharge that duty.

Another thing Dr. Melendy wants done. His idea in presenting this paper was to present it in such a way as would secure some action. What he wants done is this—that the President of this Association select some aggressive and progressive young man in the Association, who is anxious to serve the needs of the profession and humanity, and ask him to take this matter in hand, and try to keep the matter before the dentists of the State of Tennessee until Tennessee shall be well represented among contributors to this fund. His idea is that the president shall turn this matter over to some men who will take it up and do it. It should not be delegated to some of these old fellows who are about played out, but, to Dr. Cattell, or some other of the more aggressive men. I do think, gentlemen, that we are missing the greatest opportunity that we have ever had, if we lay down on this proposition. Won't you help cut the dog's tail off in one big square whack all at once?

